# Binding of the Human 14-3-3 Isoforms to Distinct Sites in the Leucine-Rich Repeat Kinase 2

**DOI:** 10.3389/fnins.2020.00302

**Published:** 2020-04-07

**Authors:** Jascha T. Manschwetus, Maximilian Wallbott, Alexandra Fachinger, Claudia Obergruber, Sabine Pautz, Daniela Bertinetti, Sven H. Schmidt, Friedrich W. Herberg

**Affiliations:** Department of Biochemistry, Institute for Biology, University of Kassel, Kassel, Germany

**Keywords:** LRRK2, 14-3-3 proteins, isoform specificity, Parkinson’s disease, phosphorylation

## Abstract

Proteins of the 14-3-3 family are well known modulators of the leucine-rich repeat kinase 2 (LRRK2) regulating kinase activity, cellular localization, and ubiquitylation. Although binding between those proteins has been investigated, a comparative study of all human 14-3-3 isoforms interacting with LRRK2 is lacking so far. In a comprehensive approach, we quantitatively analyzed the interaction between the seven human 14-3-3 isoforms and LRRK2-derived peptides covering both, reported and putative 14-3-3 binding sites. We observed that phosphorylation is an absolute prerequisite for 14-3-3 binding and generated binding patterns of 14-3-3 isoforms to interact with peptides derived from the N-terminal phosphorylation cluster (S910 and S935), the Roc domain (S1444) and the C-terminus. The tested 14-3-3 binding sites in LRRK2 preferentially were recognized by the isoforms γ and η, whereas the isoforms ϵ and especially σ showed the weakest or no binding. Interestingly, the possible pathogenic mutation Q930R in LRRK2 drastically increases binding affinity to a peptide encompassing pS935. We then identified the autophosphorylation site T2524 as a so far not described 14-3-3 binding site at the very C-terminus of LRRK2. Binding affinities of all seven 14-3-3 isoforms were quantified for all three binding regions with pS1444 displaying the highest affinity of all measured singly phosphorylated peptides. The strongest binding was detected for the combined phosphosites S910 and S935, suggesting that avidity effects are important for high affinity interaction between 14-3-3 proteins and LRRK2.

## Introduction

The leucine-rich repeat kinase 2 (LRRK2) is a large multidomain protein that is associated with familiar and sporadic Parkinson’s disease (PD) (Martin et al., 2014). In its enzymatic core region, LRRK2 harbors both a Ras of complex (Roc) GTPase domain and a kinase domain linked via the C-terminal of Roc (COR) domain. Those catalytically active domains encompass the most severe PD-associated mutations namely G2019S, I2020T in the kinase domain, and R1441C/G/H in the Roc domain (Zimprich et al., 2004; Mata et al., 2005). Additionally, Armadillo (ARM), Ankyrin (ANK), and leucine-rich repeat (LRR) domains at the N-terminus as well as a WD40 domain at the C-terminus confer structural integrity and act as scaffolds for protein-protein interactions. This complex domain architecture is assumed to regulate not only enzymatic activities of the GTPase and the kinase but also allows for spatiotemporal control throughout the cell (Gilsbach et al., 2018; Purlyte et al., 2018). Members of the Rab GTPase family were recently found to serve both as substrates as well as functional modulators of LRRK2 (Steger et al., 2016). As one of those, Rab29 recruits LRRK2 to the *trans*-Golgi network (Liu et al., 2017; Purlyte et al., 2018). Besides Rab-induced localization to membranes, LRRK2 also associates with the cytoskeleton. In this context, skein-like structures around microtubules are induced by pathogenic mutations such as R1441C or I2020T or specific kinase inhibitors (Kett et al., 2012; Blanca Ramirez et al., 2017; Schmidt et al., 2019). Members of the 14-3-3 protein family are known LRRK2 interactors enabling, both spatial control throughout the cell, as well as regulation of kinase activity (Lavalley et al., 2016).

In humans, seven 14-3-3 isoforms (β, γ, ε, ζ, η, θ, σ) have been identified which regulate activity, multimerization as well as the cellular localization of their target proteins (reviewed in Aitken, 2006). By acting as scaffolds, either homodimeric or heterodimeric, 14-3-3 proteins orchestrate numerous signaling pathways. Each 14-3-3 dimer is capable of binding to two target sequences simultaneously, thereby allowing for communication between different sites of the same polypeptide chain or even multiple proteins (Dougherty and Morrison, 2004). Despite their high sequence similarities, the seven human isoforms have different functions and interaction partners which link them to specific disease phenotypes (Dougherty and Morrison, 2004). 14-3-3 proteins have been associated with neurodegenerative diseases such as Alzheimer’s disease (ζ) and PD (γ, ϵ, ζ, θ) (Slone et al., 2015; Gu et al., 2019). In PD, pathogenic LRRK2 mutations such as G2019S alter kinase activity and can be modulated by 14-3-3 interactions (Lavalley et al., 2016). Furthermore, 14-3-3 proteins drive translocation of LRRK2 into exosomes finally leading to a secretion into the urine (Fraser et al., 2013). Another property of 14-3-3 binding to LRRK2 is protection from proteasomal degradation by inhibiting ubiquitylation and other posttranslational modifications (Zhao et al., 2015).

The binding pocket of 14-3-3 proteins is positively charged. Therefore, phosphorylation of specific sequences within target proteins can enhance affinity (Dougherty and Morrison, 2004). Yaffe and coworkers first defined sequence motifs enabling 14-3-3 interaction (Yaffe et al., 1997). One of those binding motifs, R-X-X-p[S/T]-X-P, resembles consensus sequences of AGC-kinases and is phosphorylated by the protein kinase A (PKA) with the PKA consensus sequence R-R-X-[S/T] (Kemp et al., 1977; Shabb, 2001). Our group described the PKA phosphosite S1444 (P0 position) within the Roc-domain and could demonstrate that this position enables binding of 14-3-3 proteins. 14-3-3 interaction is impaired in R1441C/G/H, one of the most common PD-related mutations, which represents the P-3 position of the PKA consensus sequence (Muda et al., 2014). This could be confirmed by Stevers et al. (2017) for 14-3-3γ. The N-terminal phosphorylation cluster, located between the ANK and LRR domain of LRRK2, encompasses the residues S910 and S935 which were identified as major 14-3-3 binding sites (Dzamko et al., 2010; Nichols et al., 2010). Another motif, defined as p[S/T]-X_1__–__2_-COOH enables binding of 14-3-3 proteins to the C-terminus of the respective interaction partner (Coblitz et al., 2006).

Here, we investigated the interaction of LRRK2 and all seven human 14-3-3 isoforms which we could confirm with pull-down experiments. Using LRRK2-derived peptides, we quantified binding to recombinant 14-3-3 proteins to discriminate isoform specificity toward the already described sites in the N-terminal phosphorylation cluster (S910 and S935) and the Roc domain (S1444). Finally, we could link the potential pathogenic mutation Q930R (Berg et al., 2005) to altered 14-3-3 binding and identified the autophosphorylation site T2524 (Pungaliya et al., 2010) at the C-terminus of LRRK2 as a so far not described 14-3-3 binding site.

## Results

**FIGURE 1:**
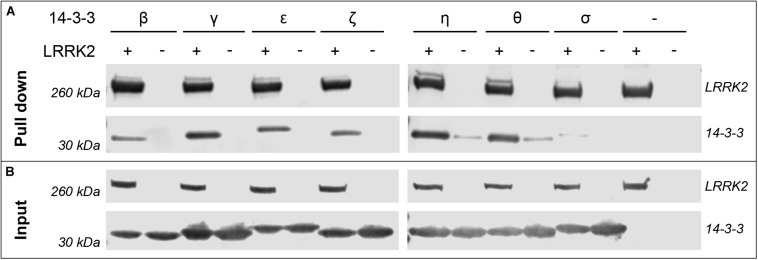
Pull-down experiments demonstrate binding of 14-3-3 isoforms to full-length LRRK2. HEK293T cells were transfected with pcDNA3.0 plasmids encoding FLAG-Strep-Strep-LRRK2 or FLAG-HA-14-3-3 as indicated with (+) and the respective isoform. (−) indicates cells that were not transfected with LRRK2 or both, 14-3-3 and LRRK2 (last lane). **(A)** 14-3-3 isoforms were co-precipitated by capturing LRRK2 with Strep-Tactin resin. Expression of proteins is shown with the input control of cell lysates **(B)**. LRRK2 and 14-3-3 proteins were detected using α-FLAG antibodies and visualized using fluorescently labeled secondary α-mouse IgG antibodies.

The interaction of 14-3-3 proteins with certain binding sites on LRRK2 has been described by several groups. Yet a comprehensive study comparing all seven human isoforms targeting these sites in LRRK2 is lacking. In an initial experiment we investigated binding of all human 14-3-3 isoforms to LRRK2 full-length protein by performing pull-down assays. For this, we co-expressed the respective 14-3-3 isoforms and FLAG-Strep-Strep-tagged LRRK2 in HEK293T cells. We were able to co-precipitate all 14-3-3 isoforms with LRRK2, although binding of σ was barely detectable (Figure 1). However, binding of 14-3-3 proteins to full-length LRRK2, as shown here, does not allow for a discrimination between distinct sites. Furthermore, the phosphostatus of the respective 14-3-3 interaction sites cannot be controlled in full-length LRRK2.

**FIGURE 2:**
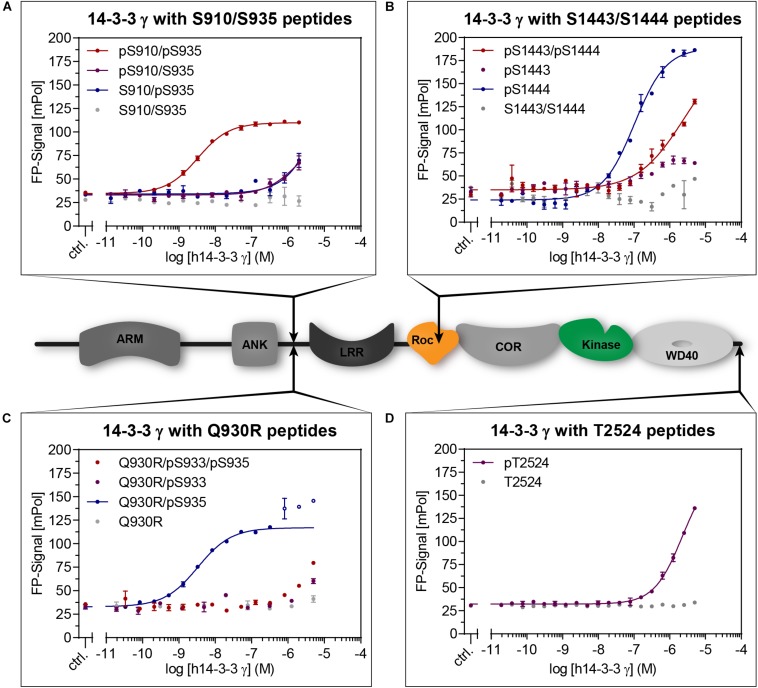
Cartoon: Domain structure of LRRK2 indicating the distinct 14-3-3 interaction sites (arrows). Binding of 14-3-3 to LRRK2-derived peptides (Table 1) was quantified with fluorescence polarization (FP). Dilution series of 14-3-3γ were measured with the indicated fluorescently labeled peptides. While non-phosphorylated peptides (gray) did not show binding, the doubly phosphorylated pS910/pS935 **(A)** demonstrated the highest affinity. From the singly phosphorylated wild type peptides, pS1444 **(B)** showed the highest affinity. The possible pathogenic mutation Q930R enables high affinity binding to pS935 **(C)**. Data points shown as circles were excluded from non-linear fits. Phosphorylated T2524 **(D)** clearly binds with micromolar affinity. All data points are means of duplicate measurements with error bars representing the standard error of mean (SEM).

We thus identified and quantified 14-3-3:LRRK2 interactions in a comprehensive study based on peptide sequences, focusing on isolated binding sites and isoform specificity. We performed fluorescence polarization (FP) assays using recombinantly expressed proteins of the seven human 14-3-3 isoforms. For this purpose, we designed fluorescently labeled LRRK2 peptides (Table 1) covering the 14-3-3 binding sites S910, S935, and S1444. Mass spectrometry studies previously revealed T2524 as a LRRK2-autophosphorylation site (Pungaliya et al., 2010). This position is located at the C-terminus of LRRK2 and thus shows similarity to the 14-3-3 binding mode III [p(S/T)-X_1__–__2_-COOH]. We therefore designed phospho- and non-phosphopeptides comprising T2524 at the very C-terminus of LRRK2.

**TABLE 1:** Peptides covering potential 14-3-3 binding sites: S910, S933, S935, S1443, S1444 and T2524.

Peptide Name	Sequence
S910	SFLVKKKSN**S**ISVGEFYRD
pS910	SFLVKKKSN**pS**ISVGEFYRD
S935	SPNLQRHSN**S**LGPIF
pS935	SPNLQRHSN**pS**LGPIF
S933	RCSPNLQRH**S**NSLGPIFDH
pS933	RCSPNLQRH**pS**NSLGPIFDH
Q930R	RCSPNL**R**RH**S**N**S**LGPIFDH
Q930R/pS933	RCSPNL**R**RH**pS**N**S**LGPIFDH
Q930R/pS935	RCSPNL**R**RH**S**N**pS**LGPIFDH
Q930R/pS933/pS935	RCSPNL**R**RH**pS**N**pS**LGPIFDH
S910/S935	KKSN **S**ISVGEFYRDAVLQRCSPNLQRHSN**S**LGPIF
pS910/S935	KKSN**pS**ISVGEFYRDAVLQRCSPNLQRHSN**S**LGPIF
S910/pS935	KKSN**S**ISVGEFYRDAVLQRCSPNLQRHSN**pS**LGPIF
pS910/pS935	KKSN**pS**ISVGEFYRDAVLQRCSPNLQRHSN**pS**LGPIF
S1443/S1444	LFNIKARA**SS**SPVILVGT
pS1443	LFNIKARA**pSS**SPVILVGT
pS1444	LFNIKARA**SpS**SPVILVGT
pS1443/pS1444	LFNIKARA**pSpS**SPVILVGT
R1441C	LFNIKA**C**A**SS**SPVILVGT
R1441C/pS1444	LFNIKA**C**A**SpS**SPVILVGT
T2524	HIEVRKELAEKMRR**T**SVE
pT2524	HIEVRKELAEKMRR**pT**SVE

*All peptides were synthesized with N-terminal fluorescein labels for FP measurements.*

15-19-mer peptide variants comprising S910, S935, S1444, or T2524 (Table 1) were first screened for binding toward 14-3-3γ (Figure 2 and Supplementary Figure S1). While no notable binding was detected for all non-phosphorylated peptides, the phosphorylated peptide pS910 demonstrated micromolar binding affinity (Supplementary Figure S1A). However, no binding was detected for the phosphorylated peptide variant pS935 (Supplementary Figure S1A). Berg et al. (2005) described the possible pathogenic mutation Q930R in LRRK2. This mutation may render S933 into a potential PKA phosphorylation site as the server-based tool NetPhos3.1 predicted with a score of 0.86 (Blom et al., 1999). A phosphorylation close to this mutated residue would subsequently generate a 14-3-3 interaction site as predicted with a consensus score of 0.71 using 14-3-3-Pred (Madeira et al., 2015). Our peptide studies revealed that neither the Q930R mutation nor the additional phosphorylation in pS933 could induce binding of 14-3-3γ (Figure 2C). In this context, we assumed that the mutation Q930R may influence binding of 14-3-3γ to pS935 (14-3-3-Pred score: 0.95). Strikingly, the Q930R mutation enabled a nanomolar affinity to pS935, which previously showed no binding in the singly phosphorylated peptides (Figure 2C, Table 2, and Supplementary Figure S1A).

**TABLE 2:** Binding affinities of human 14-3-3 isoforms toward LRRK2-derived phosphopeptides as measured by FP.

14-3-3 Isoform	β	γ	ϵ	ζ	η	θ	σ
pS910/pS935	21.1 ± 4.2	3.2 ± 0.5	12.4 ± 0.9	4.8 ± 2.2	4.8 ± 1.8	9.5 ± 2.0	60.6 ± 6.9
Q930R/pS935	29.5 ± 9.6	11.1 ± 10.7	48.5 ± 5.6	23.9 ± 0.1	13.3 ± 0.5	73.1 ± 19.7	350 ± 74
pS1444	183 ± 25	106 ± 4	>1 μM	476 ± 165	85 ± 10	195 ± 42	802 ± 261

*K_D_-values (nM) are given as means of at least three independent measurements with standard deviation (SD).*

With a *K*_*D*_-value of 106 nM, pS1444 featured the highest affinity of all singly phosphorylated wild type peptides (Figure 2B and Table 2). To investigate whether S1443, a reported PKA phosphorylation site (Muda et al., 2014), affects 14-3-3 binding, we generated another set of phosphopeptides comprising this position. While pS1443 alone did not allow for binding, the doubly phosphorylated peptide pS1443/pS1444 reduced binding to 14-3-3γ compared to pS1444 (Figure 2B). To further examine the influence of the familial PD mutation R1441C on 14-3-3 binding to the site S1444 we tested a peptide encompassing both R1441C and pS1444. Including this PD-associated mutation into the peptide (R1441C/pS1444) strongly decreased the nanomolar affinity of pS1444 alone (Supplementary Figure S1B). Again, the non-phosphorylated peptide variant encompassing the mutation R1441C displayed no binding. Finally, we tested binding to the autophosphorylation site T2524 (Pungaliya et al., 2010). Phospho and non-phosphopeptides comprising T2524 at the very C-terminus of LRRK2 were designed and demonstrated micromolar affinities for binding of 14-3-3γ to pT2524 (Figure 2D).

We next analyzed binding of 35-mers which encompass both, S910 and S935 (Figure 2A). Longer peptides were previously shown to exhibit one of the strongest affinities by enabling a dual-binding-mode of 14-3-3 dimers (Stevers et al., 2017). In line with the short peptide variant pS935 (Supplementary Figure S1A), both singly phosphorylated peptides pS910/S935 and S910/pS935 showed only weak interactions. The affinity was drastically increased to 3.2 nM for the doubly phosphorylated peptide pS910/pS935 (Table 2). 14-3-3γ, again, did not bind non-phosphorylated S910/S935 (Figure 2A).

**FIGURE 3:**
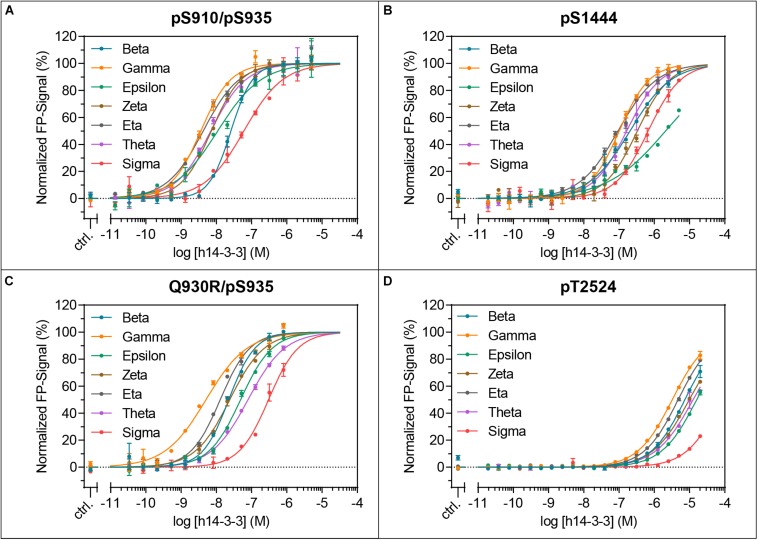
Isoform-specific binding of the LRRK2-derived peptides pS910/pS935 **(A)**, pS1444 **(B)**, Q930R/pS935 **(C)** and pT2524 **(D)** to all seven human 14-3-3 isoforms. *K*_*D*_-values were obtained with at least three independent measurements and are listed in Table 2. The resulting binding patterns are visualized in Figure 4. Data points are means of duplicate measurements with error bars representing the standard error of mean (SEM).

**FIGURE 4:**
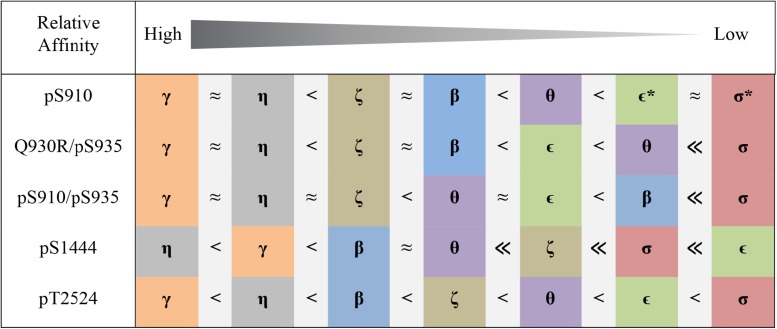
Pattern of 14-3-3 binding to distinct sites in LRRK2 based on FP experiments. Colors represent 14-3-3 isoforms as used for the binding curves (Figure 3 and Supplementary Figure S2). For the singly phosphorylated peptides Q930R/pS935 and pS1444 as well as for the doubly phosphorylated peptide pS910/pS935 *K*_*D*_-values were in the nanomolar range. Micromolar affinities were found for pS910 and pT2524 not allowing for absolute determination of *K*_*D*_-values. No binding is indicated with asterisks.

In the following analysis, binding of all seven human 14-3-3 isoforms to the above-mentioned binding sites in LRRK2 was quantified. Binding of pS910 was demonstrated for all isoforms but ϵ and σ (Supplementary Figure S2A). The highest affinities, yet in a micromolar range, were identified for γ and η followed by β and ζ while θ demonstrated the weakest binding. Under the same conditions no binding could be detected for pS935 with any 14-3-3 isoform (Supplementary Figure S2B). As expected, no non-phosphorylated control peptide bound to any isoform (Supplementary Figure S2). For the longer singly phosphorylated peptides, pS910/S935 and S910/pS935 no isoform specificity could be distinguished (Supplementary Figure S3). The doubly phosphorylated pS910/pS935 peptide enhanced binding toward all 14-3-3 isoforms to nanomolar affinities (Figure 3A). This result indicates that both phosphosites are crucial for high affinity binding. The obtained *K*_*D*_-values of the isoforms ranged from 4.8 nM for γ to 61 nM for σ (Table 2). Interestingly, the singly phosphorylated peptide Q930R/pS935 displays affinities from as high as 11 nM to as low as 350 nM (Figure 3C and Table 2). In Figure 4, the binding patterns of all 14-3-3 isoforms are visualized in a graphical overview.

pS1444 shows the highest affinities of all singly phosphorylated wild type peptides to all 14-3-3 isoforms (Figure 3B and Table 2). The isoforms γ and η exhibited the strongest binding with *K*_*D*_-values of approximately 100 nM, followed by β and θ with approximately 200 nM. While σ bound with an affinity of about 800 nM, the *K*_*D*_ of ϵ was above 1 μM. The isoform-specific binding pattern of pS1444 well compares to the one of pS910 (Figure 4). Consequently, binding of all 14-3-3 isoforms toward pS1444 was detrimentally affected by an additional phosphorylation of S1443 as shown for γ, while binding to pS1443 was not demonstrated for any isoform (Supplementary Figure S5A). All 14-3-3 isoforms interacted with R1441C/pS1444 displaying affinities in the micromolar range (Supplementary Figure S5B). However, we were not able to quantify this low affinity binding and thus could not distinguish an isoform-dependency.

We finally tested isoform-specific binding of human 14-3-3 proteins to the C-terminal autophosphorylation site using the peptide pT2524 (Figure 3D and Supplementary Figure S6). Binding affinities were in the low micromolar range, with σ showing the worst binding. The observed binding pattern again well resembled those of the singly phosphorylated peptides pS910 and pS1444 (Figure 4).

## Discussion

Malfunction of LRRK2 is correlated to PD pathogenesis suggesting that LRRK2 activity needs to be tightly controlled (Martin et al., 2014). Upstream and downstream regulators of LRRK2 have been described including kinases (e.g., PKA, casein kinase 1α), phosphatases (e.g., protein phosphatase 1), and small G-proteins (e.g., Rab29) (Lobbestael et al., 2013; Chia et al., 2014; Greggio et al., 2017; Purlyte et al., 2018). Proteins of the highly conserved 14-3-3 family represent another class of interaction partners providing control on a cellular level. 14-3-3 isoforms form homodimers as well as heterodimers by this broadening the spectrum of modulation (Jones et al., 1995). Binding of 14-3-3 proteins has been demonstrated to influence kinase activity, the phosphorylation state, and the ubiquitylation state of LRRK2 (Nichols et al., 2010; Zhao et al., 2015; Lavalley et al., 2016).

In this study we investigated the interaction of all human 14-3-3 isoforms with three different binding regions in LRRK2. We used two different approaches to investigate direct interaction between LRRK2 and the human 14-3-3 isoforms. Based on pull-down assays we qualitatively showed that all 14-3-3 isoforms except for 14-3-3σ interact with LRRK2 (Figure 1), confirming results by Nichols et al. (2010) and Li et al. (2011). LRRK2, however, is a large multidomain protein that occurs in different conformational states potentially leading to a structure-dependent protection of putative 14-3-3 binding sites. Since the accessibility of those sites on full-length protein could be limited in pull-down assays, we intended to focus our studies by reducing complexity of the interaction utilizing isolated peptide sequences. FP was therefore employed to quantitatively analyze binding affinities of 14-3-3 proteins to LRRK2-derived peptides. Our results demonstrate binding of 14-3-3 proteins in an isoform-specific manner to distinct regions in LRRK2. Within the N-terminal phosphorylation cluster we attributed the possible pathogenic mutant Q930R (Berg et al., 2005) to alter the affinity of 14-3-3 proteins toward S935. Finally, we identified phosphorylated T2524 as a so far not described interaction site at the very C-terminus of LRRK2.

### 14-3-3 Interaction With the N-Terminal Phosphorylation Cluster

The N-terminus of LRRK2 encompasses a constitutive phosphorylation cluster between the ANK and the LRR domain. In this region, the sites S860, S910, S935, S955, and S973 have been described most likely to be phosphorylated by upstream kinases (Gloeckner et al., 2010; Nichols et al., 2010; Doggett et al., 2012). Nichols et al. (2010) established S910 and S935 as major 14-3-3 binding sites which were shown later to be phosphorylated by PKA (Li et al., 2011; Muda et al., 2014).

A peptide encompassing pS910, six amino acids longer than the one employed by Stevers et al. (2017), exhibited the strongest 14-3-3 interaction site within the N-terminal phosphorylation cluster (Supplementary Figure S2A). Our studies revealed micromolar affinities for the 14-3-3 isoforms except σ and ϵ and could thereby confirm previous 14-3-3γ data of Stevers et al. (2017). Although they observed a similar binding for the peptides pS910 and pS935, we could not detect binding of our peptide pS935 to any 14-3-3 isoform (Supplementary Figure S2B).

Based on primary sequence predictions, S933 is a potential phosphorylation site which is in close proximity to the possible pathogenic mutation Q930R (Berg et al., 2005; Gloeckner et al., 2010). This mutation may render S933 into a potential PKA phosphorylation site and subsequently generates a potential 14-3-3 interaction site. However, no 14-3-3 binding was detectable for either pS933 or Q930R/pS933 (Supplementary Figure S4). Interestingly, when including the Q930R mutation into a peptide encompassing pS935, 14-3-3 affinities for all isoforms were drastically increased to nanomolar *K*_*D*_-values (Figures 2C, 3C and Table 2). This mutation may generate a possible recognition site of protein kinase B (PKB/AKT) with the consensus sequence **R**-X-R-X-X-[S/T]-**y** (*y* = hydrophobic residue) (Alessi et al., 1996; Obata et al., 2000). The impact of Q930R on 14-3-3:LRRK2 interaction may explain PD-association of this mutation.

14-3-3 proteins can also be subject to post-translational modifications such as phosphorylation or acetylation which affects the recognition of target proteins (Aitken, 2011). In this line, phosphorylation of 14-3-3γ by PAK6 weakens its interaction with LRRK2, which in turn causes dephosphorylation of pS935 (Civiero et al., 2017). These findings indicate that 14-3-3:LRRK2 interaction can be targeted by protein-protein interaction modulators in order to manipulate irregular interactions (Stevers et al., 2018b).

### 14-3-3 Interaction With the Roc Domain

Of all singly phosphorylated wild type peptides, pS1444 exhibits the highest affinity toward all 14-3-3 isoforms (Figure 3B and Table 2), which is in line with Stevers et al. (2017). S1443, adjacent to this position, is phosphorylated by PKA as well, yet incapable of 14-3-3 binding (Figure 2B and Supplementary Figure S5A; Muda et al., 2014). Compared to pS1444, the doubly phosphorylated peptide pS1443/pS1444 reduced nanomolar binding affinity to micromolar values for all isoforms (Figure 2B and Supplementary Figure S5A). We speculate that additional phosphorylation of S1443 could fine tune 14-3-3 interactions. To understand the mutual effect of S1443 and S1444 in a more physiological context, further investigations are required.

The mutational hotspot R1441 with the pathogenic mutations R1441C/G/H/S is located in close proximity to S1444 within the Roc domain (Haugarvoll and Wszolek, 2009; Mata et al., 2016; Rideout, 2017). Those mutations are known to decrease GTPase activity (Wu et al., 2019) while data on effects of R1441C on kinase activity is inconsistent as discussed by Rudenko and Cookson (2014). R1441 represents the P-3 position of a PKA consensus sequence **R-**R-X-[S/T]-y (*X* = small residue, *y* = large hydrophobic residue) with S1444 as P0 position. Binding of 14-3-3 proteins to the Roc domain depends on phosphorylation of S1444. PKA phosphorylation of this site cannot occur when R1441 is mutated (Muda et al., 2014). Comparing the peptides R1441C/pS1444 and pS1444 clearly demonstrates that even if S1444 is phosphorylated, the mutation R1441C itself decreases the affinity to all 14-3-3 isoforms at least by a factor of 30 (Supplementary Figures S1B, S5B). On a cellular level, mutations of R1441 result in distinct phenotypes. In R1441C knock-in mice both, phosphorylation of S910 and S935 as well as 14-3-3 binding, are reduced, emphasizing the relevance of this mutational hotspot (Nichols et al., 2010). R1441G induces a neurite shortening phenotype which can be reduced by overexpression of 14-3-3θ (Lavalley et al., 2016). Another effect of R1441 mutations is the accumulation of LRRK2 in cytoplasmic pools as well as filament formation around microtubules (Greggio et al., 2006; Kett et al., 2012). Interestingly, inhibiting kinase activity of LRRK2 with MLi-2 or LRRK2-IN1 induces a similar phenotype indicating a common mechanism (Dzamko et al., 2010; Schmidt et al., 2019). 14-3-3 proteins may be involved in this mechanism, since overexpression of 14-3-3β, 14-3-3γ or 14-3-3ε rescues phenotypes which are caused by R1441C mutation or by kinase inhibition (Fraser et al., 2013; Blanca Ramirez et al., 2017). Alanine substitution of the 14-3-3 binding site S1444 resulted in an increased dot-like localization but not in altered LRRK2-IN1 induced filament formation (Blanca Ramirez et al., 2017).

### 14-3-3 Interaction With the C-Terminal Helix

As we demonstrated micromolar affinities of all 14-3-3 isoforms to phosphorylated T2524, we assume a specific role of the C-terminus in regulating LRRK2 function (Figure 3D). Binding of 14-3-3 proteins occurs to three motifs in target proteins: With p[S/T]-X_1__–__2_-COOH a mode III binding motif, typically accompanied by upstream arginines, is generated at C-termini of target proteins (Coblitz et al., 2006). Although the sequence surrounding T2524 (R-R-pT-S-V-E-COOH) does not perfectly match this motif, we confirmed this autophosphorylation site as a C-terminal 14-3-3 binding site. In interleukin nine receptor alpha chain (IL-9Rα) for example, a phosphorylated serine is also located four amino acids upstream of the C-terminus, still allowing for interaction with 14-3-3 proteins (Sliva et al., 2000).

The C-terminus appears to be essential for proper LRRK2 function as Kett et al. (2012) could show that deletion of the C-terminal WD40 domain in LRRK2-I2020T results in cytosolic relocalization from a filamentous phenotype. Deletion of the last 29 residues abolished LRRK2 kinase activity, disabled microtubule association as well as interaction with 14-3-3 proteins (Rudenko et al., 2012). Rudenko and colleagues thus speculated, that the C-terminal region might tether the kinase domain in a defined conformation. Jaleel et al. (2007) could further narrow down the relevant sequence. Kinase activity was significantly reduced by deletion of the last four amino acids while removing the last seven amino acids completely abolished activity. The structure of a Roc-COR-Kinase-WD40 (RCKW) construct was just determined using cryo-EM, revealing a helical structure interacting with the kinase domain (Deniston et al., 2020). These results underline the importance of 14-3-3 binding to the C-terminus, which depends on LRRK2 autophosphorylation at T2524. Reduced autophosphorylation could result in a disturbed 14-3-3 binding to T2524. Finally, we speculate that the C-terminal helix might be autophosphorylated as a tethered substrate, allowing 14-3-3 to regulate LRRK2 function.

### Avidity Is an Important Factor for 14-3-3:LRRK2 Interaction

The combination of the low affinity peptide pS910 with the non-binding peptide pS935 resulted in the strongest 14-3-3 interaction of all tested peptides displaying affinities from 3 to 61 nM (Figure 3A and Table 2). Those avidity effects were also demonstrated by Stevers et al. (2017) for 14-3-3γ with other peptides comprising two LRRK2 phosphorylation sites. Furthermore, the group showed a contribution of the sequence between S910 and S935 on the binding event, while linkers of other doubly phosphorylated peptides had no influence (Stevers et al., 2018a). Additional evidence comes from our study, were Q930R, located in the linker, strongly increases 14-3-3 affinity to S935 (Figure 3C).

### Isoform-Specific Influence on 14-3-3:LRRK2 Interactions

In this comprehensive study we intended to investigate isoform-specific interactions of 14-3-3 proteins with the respective binding sites. Surprisingly, the resulting 14-3-3 binding patterns were very similar for the peptides pS910, Q930R/pS935, pS910/pS935, pS1444, and pT2524 (Figure 4). All 14-3-3 binding sites of LRRK2 were preferentially bound by the isoforms γ and η, in line with results by Li et al. (2011) indicating that γ and η are main interactors of full-length LRRK2. The isoforms β, θ, and ζ demonstrated intermediate binding pattern. Weakest binders are the isoforms ε and in particular σ. As shown here and by Nichols et al. (2010), 14-3-3σ, as a special member of this rather homogenous protein family, does not interact with LRRK2. This isoform is primarily found in epithelial cells (Leffers et al., 1993) and this points to a minor role of 14-3-3σ in LRRK2 regulation.

This isoform specific binding pattern is based on peptide studies, which in contrast to the pull-down assays does not reflect LRRK2 full-length protein but isolated 14-3-3 binding sites. Considering that LRRK2 is a large multidomain protein which occurs in specific conformational states, the different 14-3-3 binding sites might be dynamically accessible. Binding affinity of 14-3-3 proteins could furthermore be determined by structural properties that cannot be displayed with peptides based on the primary sequence of LRRK2 only. In this line, the LRRK2 protein structure could further affect isoform-specific binding of 14-3-3 proteins which should be addressed in future *in vivo* studies.

## Conclusion

Tight regulation of LRRK2 activities is required in order to maintain proper function since malfunction has been correlated with pathogenesis of PD (Di Maio et al., 2018). As one major regulator, 14-3-3 proteins appear to have a specific role in LRRK2 associated signaling. The phosphorylation state of LRRK2 is important for conformational control, enzymatic activities but also for protein-protein interactions allowing for spatiotemporal control. Here we demonstrate the opposing effects of the PD associated mutations Q930R and R1441C on 14-3-3 binding: in one case strengthening, in the other case weakening the respective interactions. Furthermore, we identified T2524 as a so far not described 14-3-3 binding site, highlighting the outstanding role of the LRRK2 C-terminus. In combination with the previously described sites S910, S935, and S1444, the autophosphorylation site T2524 may influence LRRK2 function. This could include cellular localization, monomer-dimer dynamics, conformational control, protein-protein interactions as well as enzymatic activities. Based on cryo-TM and cryo-EM studies of LRRK2 full-length and deletion constructs the impact of 14-3-3 proteins can finally be investigated on a structural level (Watanabe et al., 2019; Deniston et al., 2020). This will allow for a deeper understanding of how posttranslational modifications and 14-3-3 interactions affect LRRK2 biology.

## Materials and Methods

### Purification of Human 14-3-3 Isoforms

Human MBP-tagged 14-3-3 isoforms were expressed from pMAL2CX plasmids [Kilisch et al. (2016), Yuan et al. (2003)] in *E. coli* BL21DE3 cells. After induction with 0.4 M isopropyl-β-D-thiogalactoside, the protein was expressed for 4 h at room temperature. Cells were lysed in MBP-lysis buffer [50 mM Tris–HCl pH 7.5, 150 mM NaCl, 5 mM MgCl_2_, 1 mM PMSF, 1× cOmplete Protease Inhibitor EDTA-free (Roche)] using a French Pressure cell (FRENCH Press, Thermo, United States). Following centrifugation at 42,000 × *g* and 4°C for 30 min, the supernatant was transferred to a Maltose-agarose column (1.5 ml bed volume; New England Biolabs GmbH, Germany). Captured proteins were washed six times with 10 ml wash buffer (50 mM Tris–HCl pH 7.5, 150 mM NaCl, 5 mM MgCl_2_) prior to elution using wash buffer including 15 mM D-maltose. To remove the MBP tag, fusion proteins were incubated with Factor Xa (New England Biolabs GmbH, Germany), at a final concentration of 0.4 μg per 1 mg fusion protein for 24 h at RT following another 72 h at 4°C. Anion exchange chromatography was finally applied to separate residual MBP using a buffer gradient from 20 mM Tris pH 8 to 20 mM Tris pH 8 with 1 M NaCl. For this a RESOURCE Q column (GE Healthcare, United Kingdom) was utilized, employing an ÄKTApurifier (GE Healthcare, United Kingdom) or an NGC Quest Chromatography System (Bio-Rad Laboratories GmbH, Germany).

### Cell Culture, Transfection, and Purification of FLAG-Strep-Strep-Tagged LRRK2

N-terminally FLAG-Strep-Strep-tagged (FSS-) LRRK2 constructs were expressed in HEK293T cells. Plasmids (pcDNA3.0) contained the gene for full-length LRRK2 wild type (NM_198578, 1-2527). Cultivation, transfection and harvesting of cells as well as affinity purification and storage were performed as recently described (Schmidt et al., 2019).

### Strep-Tag Pull-Down

To investigate binding of all human 14-3-3 isoforms, pull-down assays were performed using the FSS-tagged full-length protein of LRRK2. For this purpose, FSS-LRRK2 was co-expressed with the respective HA-FLAG-tagged human 14-3-3 isoforms (subcloned into pcDNA3.0) in HEK293T cells. Cells of one 15 cm Ø dish were lysed in 1 ml lysis buffer [20 mM Tris pH 7.5, 150 mM NaCl, 10 mM MgCl_2_, 0.5 mM GDP, 0.5% Tween 20, 1× cOmplete Protease Inhibitor EDTA-free (Roche), PhosSTOP (Roche)]. Following a 30 min incubation at 4°C, lysates were centrifuged at 15,000 × *g* and 4°C for 20 min and subsequently the whole cell protein concentrations was determined using a Bradford assay (Bradford, 1976). Protein concentrations were adjusted to the lowest concentration to transfer equal amounts to 50 μl bed volume of equilibrated Strep-Tactin Superflow (IBA Goettingen) columns. Excessive and non-specifically bound proteins were removed by washing twice with 0.5 ml Strep-Tactin wash buffer containing 0.5 mM GDP and another five times with wash buffer containing 850 mM NaCl and 1% Tween 20. Finally, proteins were eluted and denatured in 50 μl NuPAGE LDS sample buffer (Thermo Fisher Scientific). Following Western blotting, membranes were incubated with 1:1,500 of the primary antibody ANTI-FLAG M2 (mouse, F3165, Sigma-Aldrich) over night at 4°C. To visualize target proteins, IRDye 800CW goat anti-mouse IgG secondary antibodies (LI-COR, United States) were applied at dilutions of 1:15,000 for 1 h prior to detection with an Odyssey Fc Imaging system (LI-COR, United States). Acquired images were validated using the software Image Studio Lite Version 5.2.5 (LI-COR, United States).

### Primary Amino Acid Sequence Predictions

The primary amino acid sequence of LRRK2 was obtained from UniProt Consortium (2019) and was analyzed with the webserver-based tools NetPhos3.1 and 14-3-3-Pred to predict phosphorylation sites and 14-3-3 binding sites (Blom et al., 1999; Madeira et al., 2015). Mutations were included by substituting the respective residue in the LRRK2 wild type sequence.

### Fluorescence Polarization (FP) Direct Binding Assays

FP was used to determine binding affinities of different phosphorylated and non-phosphorylated peptides derived from LRRK2 sequences (Peps4LS GmbH, Germany) toward human 14-3-3 isoforms. Direct binding assays were performed and evaluated as previously described (Muda et al., 2014; Manschwetus et al., 2019). Briefly, both dilution series of 14-3-3 isoforms ranging from final concentrations of at least 5 μM down to picomolar concentrations and dilutions of the respective fluorescein-labeled peptide (final conc. 1 nM) were prepared in FP buffer (20 mM MOPS pH 7, 150 mM NaCl, 0.005% CHAPS). Subsequently, samples were mixed in 384-well microtiter plates as duplicates (BRAND plates, pureGrade, black, BRAND GmbH & Co. KG, Germany) in a 1:1 ratio and measured using a CLARIOstar plate reader (BMG LABTECH, Germany). Two protein preparations were utilized for at least two independent replications for all measurements while high affinity binding peptides were particularly measured with a minimum of three independent replications for statistical evaluation. Data was analyzed with GraphPad Prism 6.0 (GraphPad Software, San Diego, CA, United States) by plotting obtained FP signals (mPol) against the logarithmic 14-3-3 protein concentrations. Data points represent means ± standard error of mean (SEM) of duplicates. “Ctrl.” indicates the FP signal of fluorescein-labeled peptides without 14-3-3 protein. Sigmoidal dose-response fitting was performed to define *K*_*D*_-values.

## Data Availability Statement

All datasets generated for this study are included in the article/Supplementary Material.

## Author Contributions

DB and FH conceived the project. JM, MW, DB, and FH designed research. SP performed subcloning of 14-3-3 isoforms. AF and SP designed pull-down experiments. JM, MW, and DB designed fluorescence polarization experiments. AF and CO performed experiments. JM, MW, AF, and CO analyzed data. JM and MW validated and curated data. JM, MW, DB, and SS contributed with data interpretation. JM, MW, SS, and FH wrote the manuscript. All authors have given their approval of the final version of the manuscript.

## Conflict of Interest

The authors declare that the research was conducted in the absence of any commercial or financial relationships that could be construed as a potential conflict of interest.

## Abbreviations

ANK: ankyrin repeats
ARM: armadillo repeats
Cor: C-terminal of Roc
FP: fluorescence polarization
FSS: FLAG-Strep-Strep tag
LRR: Leucine-rich repeat
LRRK2: Leucine-rich repeat kinase 2
LRRK2-IN1: LRRK2 inhibitor 1
MBP: maltose-binding protein
MLi2: Merck LRRK2 inhibitor 2
PD: Parkinson’s Disease
PKA: Protein kinase A
PKB: Protein kinase B
Roc: Ras of complex.

## References

[B1] Aitken A. (2006). 14-3-3 proteins: a historic overview. *Semin. Cancer Biol.* 16 162–172. 10.1016/j.semcancer.2006.03.005 16678438

[B2] Aitken A. (2011). Po st-translational modification of 14-3-3 isoforms and regulation of cellular function. *Semin. Cell Dev. Biol.* 22 673–680. 10.1016/j.semcdb.2011.08.003 21864699

[B3] Alessi D. R. Caudwell F. B. Andjelkovic M. Hemmings B. A. Cohen P. (1996). Molecular basis for the substrate specificity of protein kinase B; comparison with MAPKAP kinase-1 and p70 S6 kinase. *FEBS Lett.* 399 333–338. 10.1016/s0014-5793(96)01370-1 8985174

[B4] Berg D. Schweitzer K. J. Leitner P. Zimprich A. Lichtner P. Belcredi P. (2005). Type and frequency of mutations in the LRRK2 gene in familial and sporadic Parkinson’s disease. *Brain* 128 3000–3011. 10.1093/brain/awh666 16251215

[B5] Blanca Ramirez M. Lara Ordonez A. J. Fdez E. Madero-Perez J. Gonnelli A. Drouyer M. (2017). GTP binding regulates cellular localization of Parkinson’s disease-associated LRRK2. *Hum. Mol. Genet.* 26 2747–2767. 10.1093/hmg/ddx161 28453723 PMC5886193

[B6] Blom N. Gammeltoft S. Brunak S. (1999). Sequence and structure-based prediction of eukaryotic protein phosphorylation sites. *J. Mol. Biol.* 294 1351–1362. 10.1006/jmbi.1999.3310 10600390

[B7] Bradford M. M. (1976). A rapid and sensitive method for the quantitation of microgram quantities of protein utilizing the principle of protein-dye binding. *Anal. Biochem.* 72 248–254. 10.1016/0003-2697(76)90527-3 942051

[B8] Chia R. Haddock S. Beilina A. Rudenko I. N. Mamais A. Kaganovich A. (2014). Phosphorylation of LRRK2 by casein kinase 1alpha regulates trans-Golgi clustering via differential interaction with ARHGEF7. *Nat. Commun.* 5:5827. 10.1038/ncomms6827 25500533 PMC4268884

[B9] Civiero L. Cogo S. Kiekens A. Morganti C. Tessari I. Lobbestael E. (2017). PAK6 Phosphorylates 14-3-3gamma to regulate steady state phosphorylation of LRRK2. *Front. Mol. Neurosci.* 10:417. 10.3389/fnmol.2017.00417 29311810 PMC5735978

[B10] Coblitz B. Wu M. Shikano S. Li M. (2006). C-terminal binding: an expanded repertoire and function of 14-3-3 proteins. *FEBS Lett.* 580 1531–1535. 10.1016/j.febslet.2006.02.014 16494877

[B11] Deniston C. K. Salogiannis J. Mathea S. Snead D. M. Lahiri I. Donosa O. (2020). Parkinson’s Disease-linked LRRK2 structure and model for microtubule interaction. *bioRxiv* [preprint]. 10.1101/2020.01.06.895367, PMC7726071 32814344

[B12] Di Maio R. Hoffman E. K. Rocha E. M. Keeney M. T. Sanders L. H. De Miranda B. R. (2018). LRRK2 activation in idiopathic Parkinson’s disease. *Sci. Transl. Med.* 10 :eaar5429. 10.1126/scitranslmed.aar5429 PMC6344941 30045977

[B13] Doggett E. A. Zhao J. Mork C. N. Hu D. Nichols R. J. (2012). Phosphorylation of LRRK2 serines 955 and 973 is disrupted by Parkinson’s disease mutations and LRRK2 pharmacological inhibition. *J. Neurochem.* 120 37–45. 10.1111/j.1471-4159.2011.07537.x 22004453

[B14] Dougherty M. K. Morrison D. K. (2004). Unlocking the code of 14-3-3. *J. Cell Sci.* 117 1875–1884. 10.1242/jcs.01171 15090593

[B15] Dzamko N. Deak M. Hentati F. Reith A. D. Prescott A. R. Alessi D. R. (2010). Inhibition of LRRK2 kinase activity leads to dephosphorylation of Ser(910)/Ser(935), disruption of 14-3-3 binding and altered cytoplasmic localization. *Biochem. J.* 430 405–413. 10.1042/BJ20100784 20659021 PMC3631100

[B16] Fraser K. B. Moehle M. S. Daher J. P. Webber P. J. Williams J. Y. Stewart C. A. (2013). LRRK2 secretion in exosomes is regulated by 14-3-3. *Hum. Mol. Genet*, 22 4988–5000. 10.1093/hmg/ddt346 23886663 PMC3836478

[B17] Gilsbach B. K. Eckert M. Gloeckner C. J. (2018). Regulation of LRRK2: insights from structural and biochemical analysis. *Biol. Chem.* 399 637–642. 10.1515/hsz-2018-0132 29894291

[B18] Gloeckner C. J. Boldt K. Von Zweydorf F. Helm S. Wiesent L. Sarioglu H. (2010). Phosphopeptide analysis reveals two discrete clusters of phosphorylation in the N-terminus and the Roc domain of the Parkinson-disease associated protein kinase LRRK2. *J. Proteome Res.* 9 1738–1745. 10.1021/pr9008578 20108944

[B19] Greggio E. Bubacco L. Russo I. (2017). Cross-talk between LRRK2 and PKA: implication for Parkinson;s disease? *Biochem. Soc. Trans.* 45 261–267. 10.1042/BST20160396 28202680

[B20] Greggio E. Jain S. Kingsbury A. Bandopadhyay R. Lewis P. Kaganovich A. (2006). Kinase activity is required for the toxic effects of mutant LRRK2/dardarin. *Neurobiol. Dis.* 23 329–341. 10.1016/j.nbd.2006.04.001 16750377

[B21] Gu Q. Cuevas E. Raymick J. Kanungo J. Sarkar S. (2019). Downregulation of 14-3-3 Proteins in Alzheimer’s Disease. *Mol Neurobiol.* 57 32–40. 10.1007/s12035-019-01754-y 31487003

[B22] Haugarvoll K. Wszolek Z. K. (2009). Clinical features of LRRK2 parkinsonism. *Park. Relat. Disord.* 15 S205–S208. 10.1016/S1353-8020(09)70815-6 20082991

[B23] Jaleel M. Nichols R. J. Deak M. Campbell D. G. Gillardon F. Knebel A. (2007). LRRK2 phosphorylates moesin at threonine-558: characterization of how Parkinson’s disease mutants affect kinase activity. *Biochem. J* 405 307–317. 10.1042/bj20070209 17447891 PMC1904520

[B24] Jones D. H. Ley S. Aitken A. (1995). Isoforms of 14-3-3 protein can form homo- and heterodimers in vivo and in vitro: implications for function as adapter proteins. *FEBS Lett.* 368 55–58. 10.1016/0014-5793(95)00598-4 7615088

[B25] Kemp B. E. Graves D. J. Benjamini E. Krebs E. G. (1977). Role of multiple basic residues in determining the substrate specificity of cyclic AMP-dependent protein kinase. *J. Biol. Chem.* 252 4888–4894. 194899

[B26] Kett L. R. Boassa D. Ho C. C. Rideout H. J. Hu J. Terada M. (2012). LRRK2 Parkinson disease mutations enhance its microtubule association. *Hum. Mol. Genet.* 21 890–899. 10.1093/hmg/ddr526 22080837 PMC3263991

[B27] Kilisch M. Lytovchenko O. Arakel E. C. Bertinetti D. Schwappach B. (2016). A dual phosphorylation switch controls 14-3-3-dependent cell surface expression of TASK-1. *J. Cell Sci.* 129 831–842. 10.1242/jcs.180182 26743085 PMC4760375

[B28] Lavalley N. J. Slone S. R. Ding H. West A. B. Yacoubian T. A. (2016). 14-3-3 Proteins regulate mutant LRRK2 kinase activity and neurite shortening. *Hum. Mol. Genet.* 25 109–122. 10.1093/hmg/ddv453 26546614 PMC4690493

[B29] Leffers H. Madsen P. Rasmussen H. H. Honore B. Andersen A. H. Walbum E. (1993). Molecular cloning and expression of the transformation sensitive epithelial marker stratifin. A member of a protein family that has been involved in the protein kinase C signalling pathway. *J. Mol. Biol.* 231 982–998. 10.1006/jmbi.1993.1346 8515476

[B30] Li X. Wang Q. J. Pan N. Lee S. Zhao Y. Chait B. T. (2011). Phosphorylation-dependent 14-3-3 binding to LRRK2 is impaired by common mutations of familial Parkinson’s disease. *PLoS One* 6:e17153. 10.1371/journal.pone.0017153 21390248 PMC3046972

[B31] Liu Z. Bryant N. Kumaran R. Beilina A. Abeliovich A. Cookson M. R. (2017). LRRK2 phosphorylates membrane-bound Rabs and is activated by GTP-bound Rab7L1 to promote recruitment to the trans-Golgi network. *Hum. Mol. Genet.* 27 385–395. 10.1093/hmg/ddx410 29177506 PMC5886198

[B32] Lobbestael E. Zhao J. Rudenko I. N. Beylina A. Gao F. Wetter J. (2013). Identification of protein phosphatase 1 as a regulator of the LRRK2 phosphorylation cycle. *Biochem. J.* 456 119–128. 10.1042/BJ20121772 23937259 PMC5141581

[B33] Madeira F. Tinti M. Murugesan G. Berrett E. Stafford M. Toth R. (2015). 14-3-3-Pred: improved methods to predict 14-3-3-binding phosphopeptides. *Bioinformatics* 31 2276–2283. 10.1093/bioinformatics/btv133 25735772 PMC4495292

[B34] Manschwetus J. T. Bendzunas G. N. Limaye A. J. Knape M. J. Herberg F. W. Kennedy E. J. (2019). A stapled peptide mimic of the pseudosubstrate inhibitor PKI inhibits protein kinase A. *Molecules* 24:1567. 10.3390/molecules24081567 31009996 PMC6514771

[B35] Martin I. Kim J. W. Dawson V. L. Dawson T. M. (2014). LRRK2 pathobiology in Parkinson’s disease. *J. Neurochem.* 131 554–565. 10.1111/jnc.12949 25251388 PMC4237709

[B36] Mata I. F. Davis M. Y. Lopez A. N. Dorschner M. O. Martinez E. Yearout D. (2016). The discovery of LRRK2 p.*R1441S*, a novel mutation for Parkinson’s disease, adds to the complexity of a mutational hotspot. *Am. J. Med. Genet. B Neuropsychiatr. Genet.* 171 925–930. 10.1002/ajmg.b.32452 27111571 PMC5028305

[B37] Mata I. F. Kachergus J. M. Taylor J. P. Lincoln S. Aasly J. Lynch T. (2005). Lrrk2 pathogenic substitutions in Parkinson’s disease. *Neurogenetics* 6 171–177. 16172858 10.1007/s10048-005-0005-1

[B38] Muda K. Bertinetti D. Gesellchen F. Hermann J. S. Von Zweydorf F. Geerlof A. (2014). Parkinson-related LRRK2 mutation R1441C/G/H impairs PKA phosphorylation of LRRK2 and disrupts its interaction with 14-3-3. *Proc. Natl. Acad. Sci. U.S.A.* 111 E34–E43. 10.1073/pnas.1312701111 24351927 PMC3890784

[B39] Nichols R. J. Dzamko N. Morrice N. A. Campbell D. G. Deak M. Ordureau A. (2010). 14-3-3 binding to LRRK2 is disrupted by multiple Parkinson’s disease-associated mutations and regulates cytoplasmic localization. *Biochem. J.* 430 393–404. 10.1042/BJ20100483 20642453 PMC2932554

[B40] Obata T. Yaffe M. B. Leparc G. G. Piro E. T. Maegawa H. Kashiwagi A. (2000). Peptide and protein library screening defines optimal substrate motifs for AKT/PKB. *J. Biol. Chem.* 275 36108–36115. 10.1074/jbc.m005497200 10945990

[B41] Pungaliya P. P. Bai Y. Lipinski K. Anand V. S. Sen S. Brown E. L. (2010). Identification and characterization of a leucine-rich repeat kinase 2 (LRRK2) consensus phosphorylation motif. *PLoS One* 5:e13672. 10.1371/journal.pone.0013672 21060682 PMC2965117

[B42] Purlyte E. Dhekne H. S. Sarhan A. R. Gomez R. Lis P. Wightman M. (2018). Rab29 activation of the Parkinson’s disease-associated LRRK2 kinase. *EMBO J.* 37 1–18. 10.15252/embj.201798099 29212815 PMC5753036

[B43] Rideout H. J. (2017). *Leucine-Rich Repeat Kinase 2 (LRRK2).* Berlin: Springer.

[B44] Rudenko I. N. Cookson M. R. (2014). Heterogeneity of leucine-rich repeat kinase 2 mutations: genetics, mechanisms and therapeutic implications. *Neurotherapeutics* 11 738–750. 10.1007/s13311-014-0284-z 24957201 PMC4391379

[B45] Rudenko I. N. Kaganovich A. Hauser D. N. Beylina A. Chia R. Ding J. (2012). The G2385R variant of leucine-rich repeat kinase 2 associated with Parkinson’s disease is a partial loss-of-function mutation. *Biochem. J/* 446 99–111. 10.1042/BJ20120637 22612223 PMC4667980

[B46] Schmidt S. H. Knape M. J. Boassa D. Mumdey N. Kornev A. P. Ellisman M. H. (2019). The dynamic switch mechanism that leads to activation of LRRK2 is embedded in the DFGpsi motif in the kinase domain. *Proc. Natl. Acad. Sci. U.S.A.* 116 14979–14988. 10.1073/pnas.1900289116 31292254 PMC6660771

[B47] Shabb J. B. (2001). Physiological substrates of cAMP-dependent protein kinase. *Chem. Rev.* 101 2381–2411. 11749379 10.1021/cr000236l

[B48] Sliva D. Gu M. Zhu Y. X. Chen J. Tsai S. Du X. (2000). 14-3-3zeta interacts with the alpha-chain of human interleukin 9 receptor. *Biochem. J.* 345(Pt 3), 741–747. 10642536 PMC1220812

[B49] Slone S. R. Lavalley N. Mcferrin M. Wang B. Yacoubian T. A. (2015). Increased 14-3-3 phosphorylation observed in Parkinson’s disease reduces neuroprotective potential of 14-3-3 proteins. *Neurobiol. Dis.* 79 1–13. 10.1016/j.nbd.2015.02.032 25862939 PMC4458424

[B50] Steger M. Tonelli F. Ito G. Davies P. Trost M. Vetter M. (2016). Phosphoproteomics reveals that Parkinson’s disease kinase LRRK2 regulates a subset of Rab GTPases. *Elife* 5. e12813. 10.7554/eLife.12813 26824392 PMC4769169

[B51] Stevers L. M. De Vink P. J. Ottmann C. Huskens J. Brunsveld L. (2018a). A thermodynamic model for multivalency in 14-3-3 protein-protein interactions. *J. Am. Chem. Soc.* 140 14498–14510. 10.1021/jacs.8b09618 30296824 PMC6213025

[B52] Stevers L. M. De Vries R. M. Doveston R. G. Milroy L. G. Brunsveld L. Ottmann C. (2017). Structural interface between LRRK2 and 14-3-3 protein. *Biochem. J.* 474 1273–1287. 10.1042/BCJ20161078 28202711

[B53] Stevers L. M. Sijbesma E. Botta M. Mackintosh C. Obsil T. Landrieu I. (2018b). Modulators of 14-3-3 protein-protein interactions. *J. Med. Chem.* 61 3755–3778. 10.1021/acs.jmedchem.7b00574 28968506 PMC5949722

[B54] UniProt Consortium (2019). UniProt: a worldwide hub of protein knowledge. *Nucleic Acids Res.* 47 D506–D515. 10.1093/nar/gky1049 30395287 PMC6323992

[B55] Watanabe R. Buschauer R. Boehning J. Audagnotto M. Lasker K. Lu T. W. (2019). The in situ structure of Parkinson’s disease-linked LRRK2. *BioRxiv* [preprint]. 10.1101/837203 PMC7869717 32783917

[B56] Wu C. X. Liao J. Park Y. Reed X. Engel V. A. Hoang N. C. (2019). Parkinson’s disease-associated mutations in the GTPase domain of LRRK2 impair its nucleotide-dependent conformational dynamics. *J. Biol. Chem.* 294 5907–5913. 10.1074/jbc.RA119.007631 30796162 PMC6463707

[B57] Yaffe M. B. Rittinger K. Volinia S. Caron P. R. Aitken A. Leffers H. (1997). The structural basis for 14-3-3:phosphopeptide binding specificity. *Cell* 91 961–971. 10.1016/s0092-8674(00)80487-0 9428519

[B58] Yuan H. Michelsen K. Schwappach B. (2003). 14-3-3 dimers probe the assembly status of multimeric membrane proteins. *Curr. Biol.* 13 638–646. 10.1016/s0960-9822(03)00208-2 12699619

[B59] Zhao J. Molitor T. P. Langston J. W. Nichols R. J. (2015). LRRK2 dephosphorylation increases its ubiquitination. *Biochem. J.* 469 107–120. 10.1042/BJ20141305 25939886 PMC4613513

[B60] Zimprich A. Biskup S. Leitner P. Lichtner P. Farrer M. Lincoln S. (2004). Mutations in LRRK2 cause autosomal-dominant parkinsonism with pleomorphic pathology. *Neuron* 44 601–607. 10.1016/j.neuron.2004.11.005 15541309

