# Supplementary material for: Binding of the Human 14-3-3 Isoforms to Distinct Sites in the Leucine-Rich Repeat Kinase 2
Source: Front Neurosci. 2020 Apr 7;14:302. doi: 10.3389/fnins.2020.00302 (PMC7155755; doi:10.3389/fnins.2020.00302)
Supplement: Supplementary file 1 [file Table_1.pdf]

frontiers

Supplementary Material

1 Supplementary Data

All data points shown are means of duplicate measurements with error bars representing the standard error of mean (SEM).

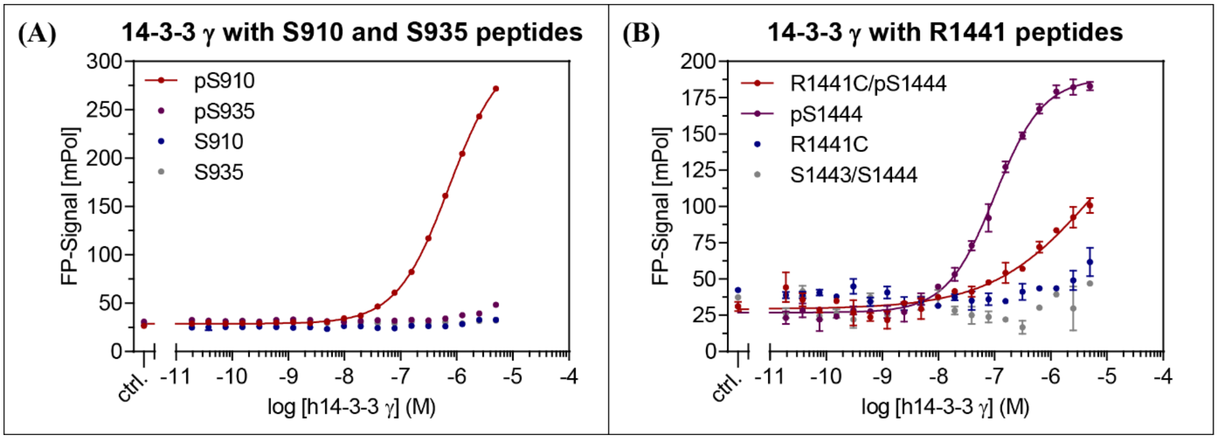

SUPPLEMENTARY FIGURE S1: Binding of 14-3-3 to LRRK2-derived peptides (**Table 1**) was quantified with fluorescence polarization (FP). Dilution series of 14-3-3γ were measured with the indicated fluorescently labeled peptides as described in **Figure 2**. Non-phosphorylated peptides (gray), pS935 **(A)** and R1441C **(B)** did not bind, while the peptides pS910 **(A)** and R1441C/pS1444 **(B)** showed micromolar affinities. pS1444 **(B)** enabled the strongest binding.

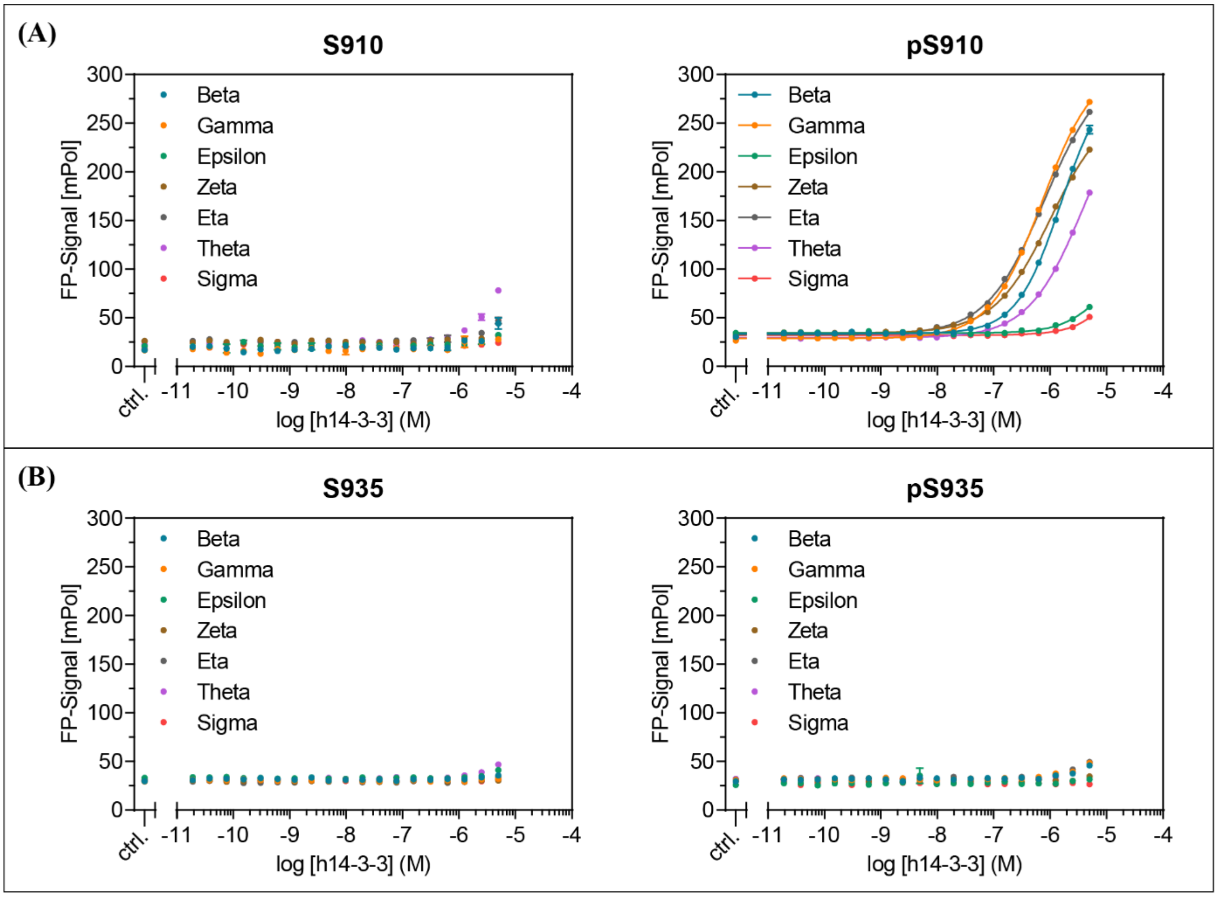

SUPPLEMENTARY FIGURE S2: Binding of LRRK2-derived peptides encompassing the sites S910 **(A)** or S935 **(B)** to all human 14-3-3 isoforms (left: non-phosphorylated, right: phosphorylated). pS910 was the only peptide showing micromolar affinity for all isoforms but ϵ and σ.

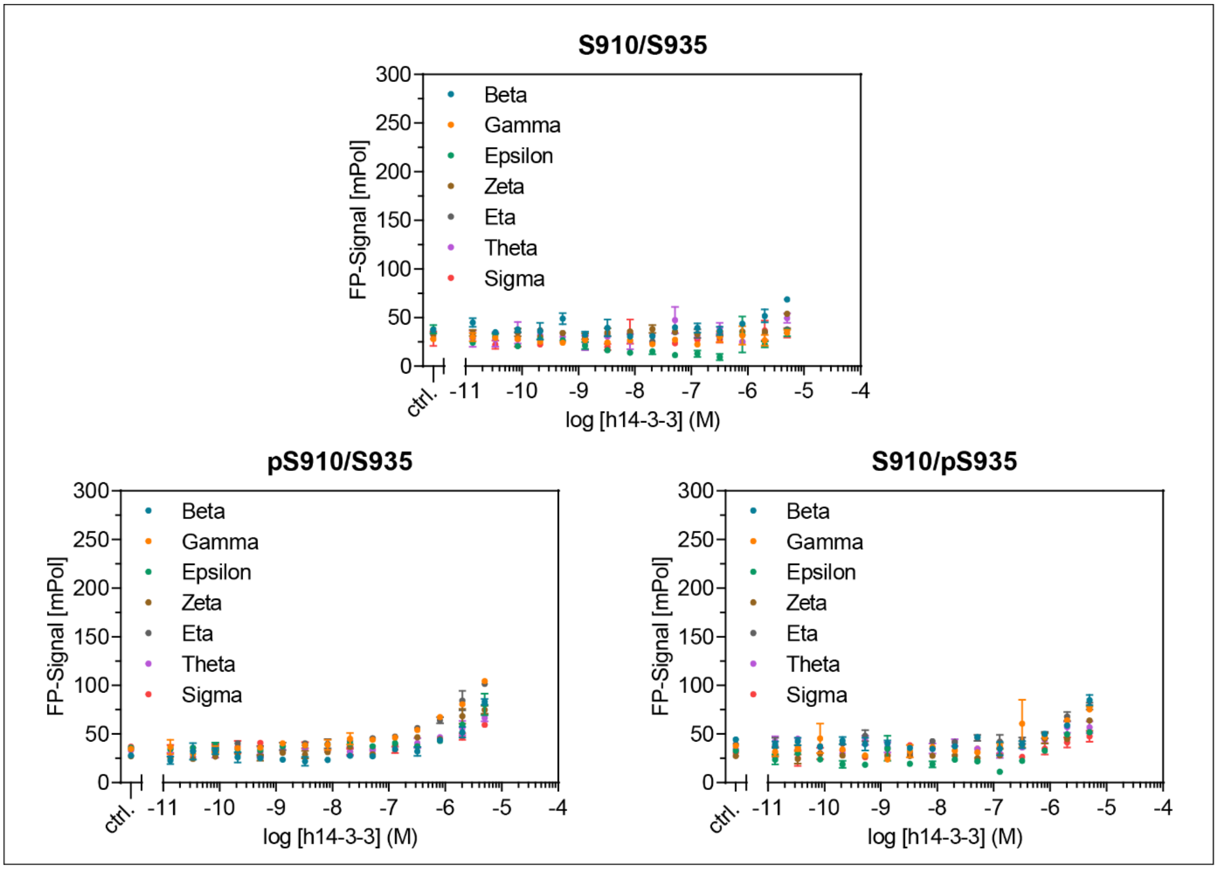

SUPPLEMENTARY FIGURE S3: FP measurements of the long peptides encompassing both 14-3-3 binding sites S910 and S935 of LRRK2. Weak interactions were detected for the singly phosphorylated peptides pS910/S935 and S910/pS935 compared to the non-phosphorylated peptide S910/S935 toward all human 14-3-3 isoforms.

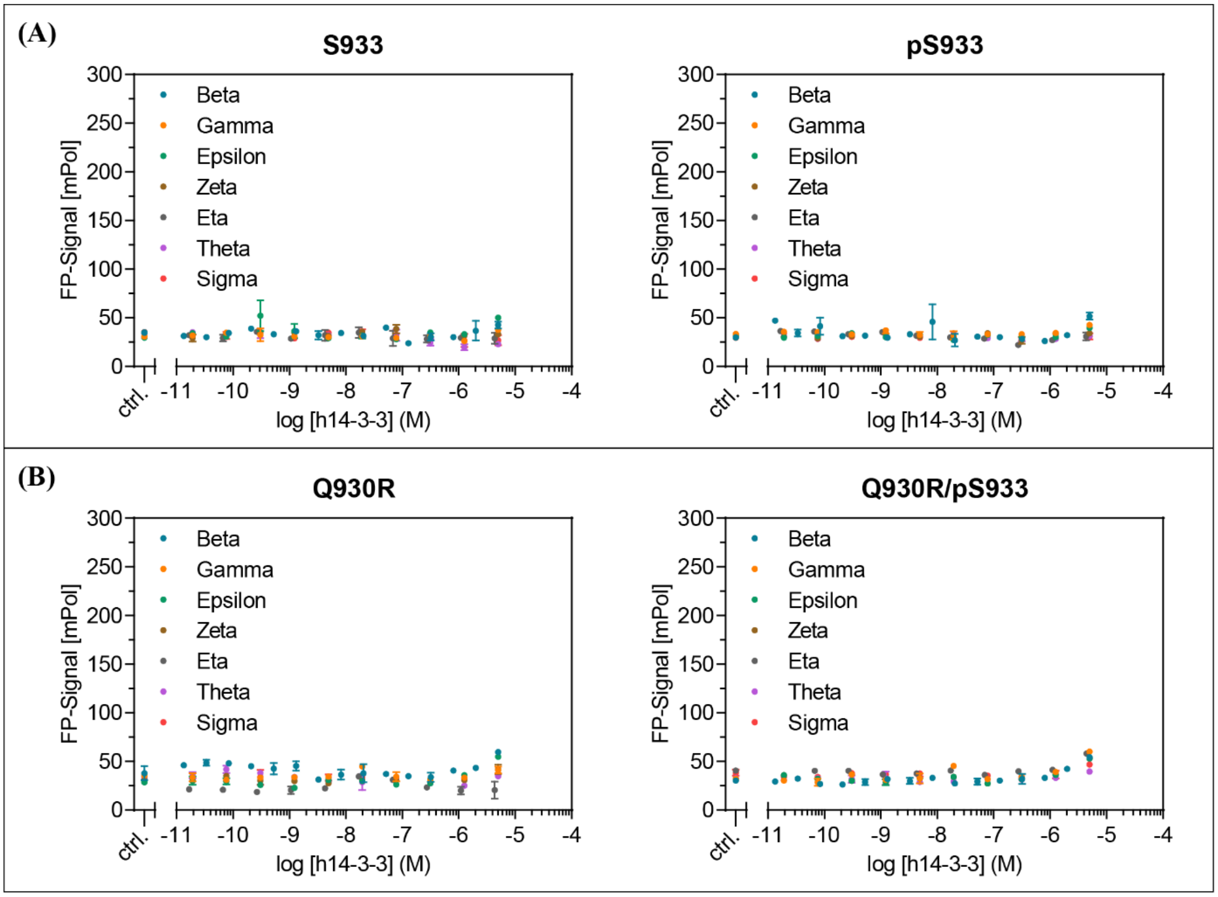

SUPPLEMENTARY FIGURE S4: Phosphorylations in peptides comprising either the site S933 alone **(A)** or including the possible pathogenic mutation Q930R **(B)** did not enable binding of any 14-3-3 isoform.

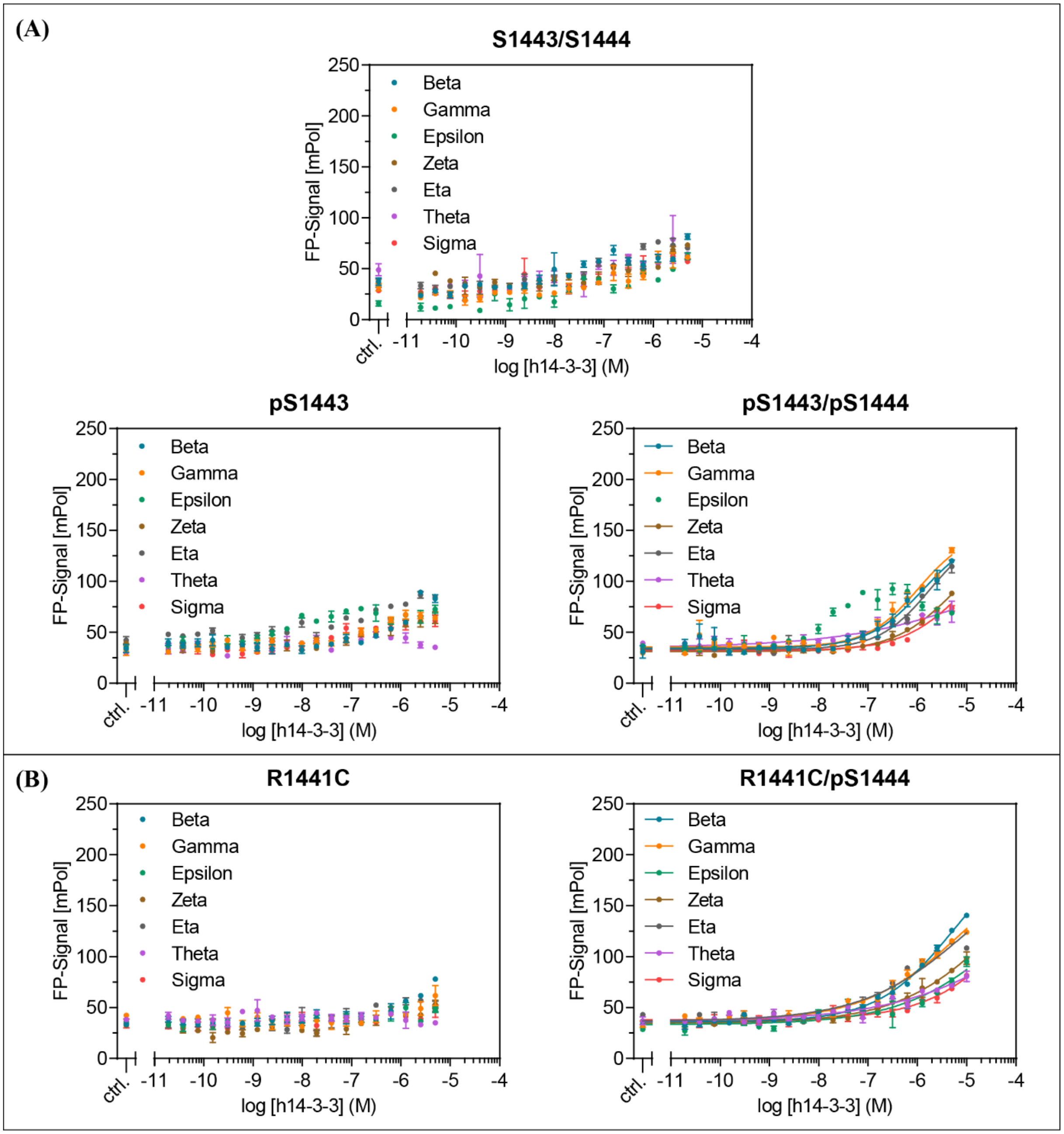

SUPPLEMENTARY FIGURE S5: Binding all human 14-3-3 isoforms to LRRK2-derived peptides encompassing the phosphorylation site S1444 in the Roc domain. The influence of S1443 on 14-3-3 binding was investigated in **(A)**. The non-phosphorylated peptide S1443/S1444 (upper panel) and pS1443 showed no detectable binding. Combination of the phosphorylation sites in pS1443/pS1444 resulted in micromolar affinities. A peptide including the pathogenic mutation R1441C alone **(B, left)** did not bind any of the 14-3-3 isoforms and showed micromolar affinities when combined with pS1444 **(B, right)**, yet reduced compared to pS1444 (**Figure 3B**). Top values of non-linear fits were constrained to 150 mPol for pS1443/pS1444 and to 215 mPol for R1441C/pS1444. The binding curve of ϵ pS1443/pS1444 was not fitted due to an unusual binding behavior **(A)**.

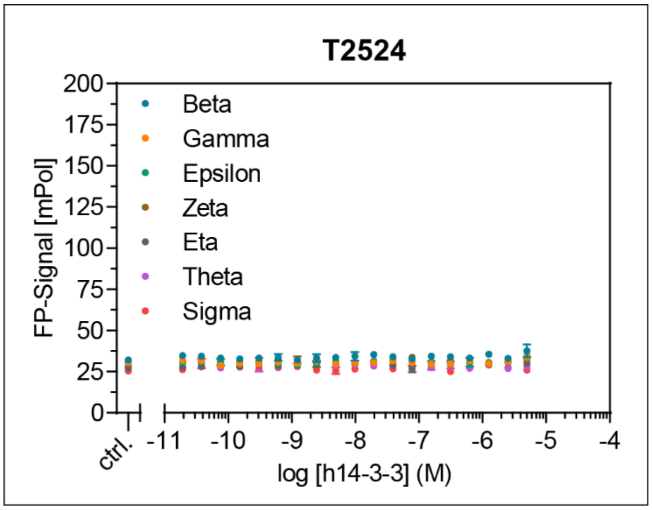

SUPPLEMENTARY FIGURE S6: Binding of all human 14-3-3 isoforms to the peptide T2524, derived from the LRRK2 C-terminus, was abolished compared to pT2524 (**Figure 3D**).
